# Adult onset aqueductal stenosis may become symptomatic due to deep white matter ischemia.

**DOI:** 10.1186/2045-8118-12-S1-O26

**Published:** 2015-09-18

**Authors:** William Guerin Bradley, Mohammed Abdihalim, Abdulrachman Almutairi

**Affiliations:** 1 UCSD, USA

## Introduction

The term “Aqueductal stenosis” generally implies a congenital etiology which may or may not be compatible with life. Those that survive probably have an aqueductal membrane with a pinhole, allowing at least some CSF through. These patients may require an alternate CSF drainage pathway via the extracellular space (ECS) of the brain as occurs in children with tectal gliomas. It is likely that there is a “second hit” in addition to this congenital condition that leads to symptom onset in late adulthood. We hypothesize that the onset of deep white matter ischemia in late adulthood increases resistance to CSF flow through the extracellular space of the brain, contributing to the onset of symptoms of aqueductal stenosis in late adulthood.

## Methods

Retrospective review of our database searching for the keywords of “aqueductal stenosis” from August 2009-April 2015, yielded 15 cases of adult onset aqueductal stenosis confirmed on a midsagittal FIESTA or CISS image. The apparent diffusion coefficient (ADC) in four regions in the centrum semiovale were measured as a surrogate of the amount of water in the ECS in patients with aqueductal stenosis and in controls with the same amount of deep white matter ischemia (DWMI).

## Results

ADC measurements performed in the centrum semiovale are significantly higher in patients with aqueductal stenosis than in controls (P < .01), controlling for the same degree of DWMI. This indicates that there is increased fluid in the ECS of the brain of the aqueductal stenosis cases, supporting the hypothesis that the parallel pathway for CSF egress is via the ECS.

## Conclusion

Increasing resistance to CSF outflow through the ECS of the brain due to deep white matter ischemia in late adulthood may contribute to the development of symptoms in adult onset aqueductal stenosis.
